# Pregnancy in women with vascular Ehlers-Danlos syndrome: a case series from the registry of pregnancy and cardiac disease (ROPAC) III

**DOI:** 10.1016/j.ijcchd.2026.100671

**Published:** 2026-03-28

**Authors:** P.N.J. Peters, J.A. van der Zande, S.K. Prakash, J. Harris, E. Troost, D. Tobler, B.J. Bouma, J.W. Roos-Hesselink, the ROPAC investigators

**Affiliations:** a Department of Cardiology, Erasmus MC, University Medical Center Rotterdam, Rotterdam, the Netherlands; b Department of Obstetrics and Gynaecology, Erasmus MC, University Medical Center Rotterdam, Rotterdam, the Netherlands; c Department of Internal Medicine, McGovern Medical School, The University of Texas Health Science Center, Houston, TX, USA; d Department of Cardiology, North Shore Private Hospital, St Leonards, Sydney, Australia; e Department of Cardiology, University Hospital Leuven, Leuven, Belgium; f Department of Cardiology, University Hospital Basel, Basel, Switzerland; g Department of Cardiology, Amsterdam University Medical Center, Amsterdam, the Netherlands

**Keywords:** Case series, Vascular Ehlers-Danlos syndrome, Pregnancy, ROPAC

## Abstract

**Background:**

Vascular Ehlers-Danlos syndrome (vEDS) is a rare connective tissue disorder associated with high pregnancy-related risks. Current guidelines consider vEDS an mWHO IV high-risk pregnancy, but data are limited and often retrospective.

**Methods:**

This prospective case series reports pregnancy outcomes and aortic measurements in six women with confirmed or suspected vEDS enrolled in the ROPAC III registry.

**Results:**

No maternal deaths or aortic dissections occurred. One fetal death was reported. Obstetric complications were common, including preterm birth and hemorrhage. Most women delivered via cesarean section. No significant aortic growth was observed during pregnancy.

**Conclusions:**

In women diagnosed with vEDS prior to pregnancy, the absence of life-threatening vascular events suggests pregnancy may be safer than previously reported. These findings support more individualized counseling and underscore the need for further prospective studies.

## Highlights

• Pregnancies in women known with vascular Ehlers-Danlos syndrome pre-pregnancyseem lower risk than previously thought.
• Pre-pregnancy counseling and genotyping in women with vascular Ehlers-Danlos syndrome remains crucial.
• Cardiac and obstretric complications do occur during pregnancy in women with vascular Ehlers-Danlos syndrome.

## Graphical abstract

**Figure:**
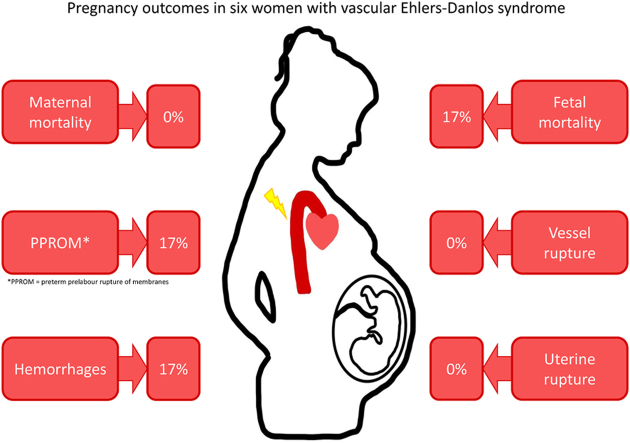

## 1 Introduction

Vascular Ehlers-Danlos syndrome (vEDS) is a rare inherited disorder resulting from a dominant pathogenic variant in the *COL3A1* gene, which encodes the proalpha-1 chains of type III collagen [1]. Collagen type III is a protein responsible for structural integrity of the skin, vessel walls and viscera [2], which explains increased bruising, arterial and bowel complications, and uterine, cervical and vaginal fragility in patients with vEDS [3]. The risk of complications is thought to vary depending on the type of pathogenic variant in *COL3A1* [4,5].

The significance of the problems these patients face is highlighted during pregnancy and the postpartum period. In addition to hormonal changes, the increased heart rate and blood pressure augment the risk of vessel rupture. Also, contractions induce stress on the structurally weaker uterus [6]. Complications have been described in up to 50% of the pregnancies including aortic dissection, as well as premature rupture of membranes (PPROM), uterine rupture, severe perineal tears and hemorrhages. The pregnancy-related mortality rate is around 5%, which is approximately 300 times higher than the general pregnant population [4,7]. However, previous research has found that overall survival rates are not altered by pregnancy [8].

Due to the rarity of this disease, patients are often unaware of their condition when they become pregnant. That is why many case reports describe the first presentation of vEDS due to complications during pregnancy or postpartum, inducing selection bias of more severe cases, probably overestimating the risks [[9], [10], [11], [12]]. No prospective studies have been conducted before and data on aortic measurements are lacking.

To address gaps in knowledge, the EURObservational Research Programme (EORP) of the ESC initiated the ROPAC III in 2018, which was designed to be a global prospective registry focusing on women with prosthetic valves and/or aortic pathology, diagnosed before pregnancy. In this case series, we report detailed pregnancy outcomes and echocardiographic data of six women with vEDS.

## 2 Methods

ROPAC is an international, prospective, observational registry initiated in 2007 by the ESC as a part of the EORP to study pregnant women with structural heart disease. In 2018, ROPAC III commenced focusing on pregnancies in women known to have one or more prosthetic valves and/or known aortic pathology or genetic conditions associated with aortic pathology, diagnosed before pregnancy. Pregnancies were prospectively included until April 2023.

Main outcomes were major cardiac event or maternal mortality (MACE) during pregnancy, including aortic dissection, rapid aortic growth (>3 mm during pregnancy) of any aortic segment, any new diagnosis of congestive heart failure or arrhythmia, thromboembolic complications (thrombosis/pulmonary embolism/stroke), or endocarditis. Other outcomes included obstetric, fetal and delivery complications, breastfeeding and echocardiographic data. Aortic measurements were reported by the local investigator.

**Fig. 1:**
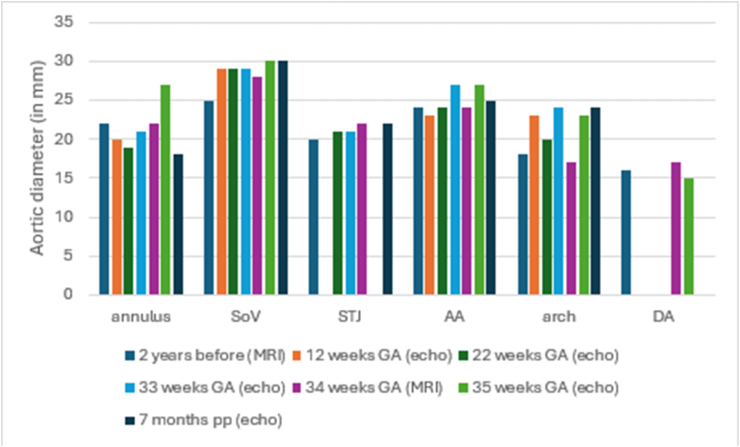
Patient 1 imaging data of aortic measurements stratified per aortic segment AA, ascending aorta; DA, descending aorta; GA, gestational age; pp, postpartum; SoV, sinus of Valsalva; STJ, sino-tubular junction.

**Fig. 2:**
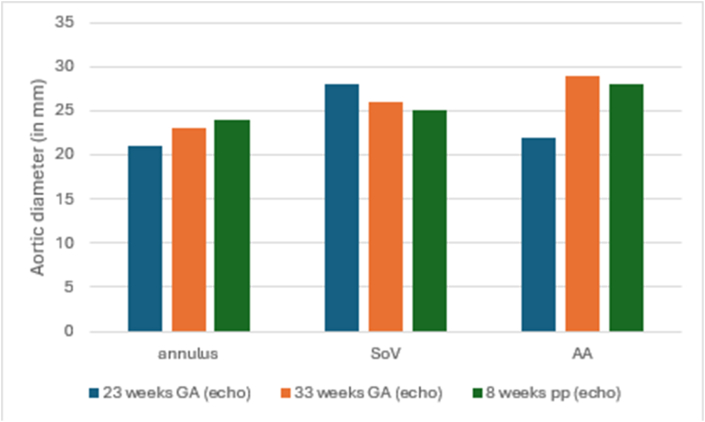
Patient 2 imaging data of aortic measurements stratified per aortic segment AA, ascending aorta; DA, descending aorta; GA, gestational age; pp, postpartum; SoV, sinus of Valsalva.

**Fig. 3:**
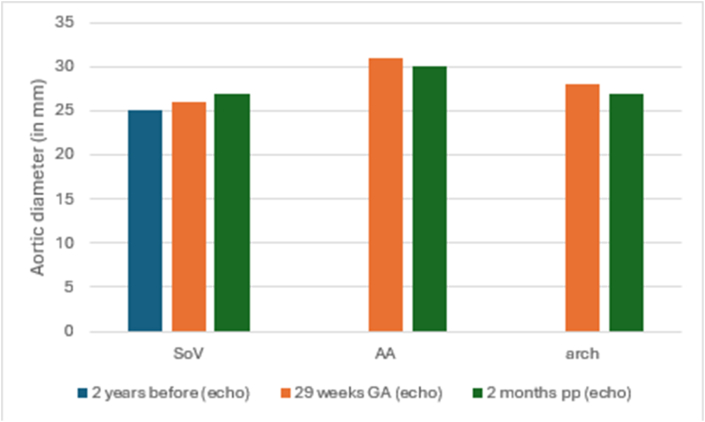
Patient 3 imaging data of aortic measurements stratified per aortic segment AA, ascending aorta; GA, gestational age; pp, postpartum; SoV, sinus of Valsalva.

**Fig. 4:**
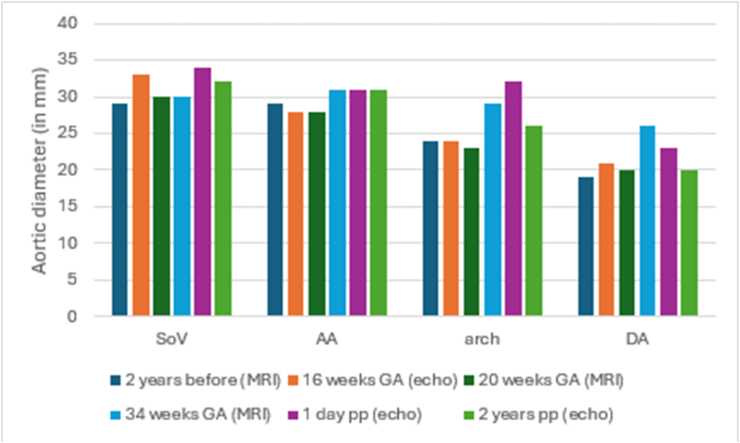
Patient 4 imaging data of aortic measurements stratified per aorta segment AA, ascending aorta; DA, descending aorta; GA, gestational age; pp, postpartum; SoV, sinus of V.alsalva.

Linear mixed-effects models were used to analyse the imaging data of all patients combined (Fig. 1, Fig. 2, Fig. 3, Fig. 4). Statistical analysis was performed using IBM SPSS Statistics version 25.0. The examinations were used as the independent variable, aortic diameters were used as the dependent variable and random effects were used to account for multiple exams per woman.

## 3 Results

Detailed information from all patients is provided in Table 1, Table 2.

**Table 1:** Detailed information pre-pregnancy of all patients.

	Age	BMI	NYHA class	LMIC/HIC	Gravida & Para	Counseling	Type of PV	History of arterial pathology	Family history of aortic pathology	History	Max. diameter before	Medication before pregnancy
**Patient 1**	32	18	I	HIC	G2P0	Cardiological	Substitution C.1258G > A (P.GLY420SER)	Negative	Positive	Therapeutic abortion (4 years prior) Miscarriage (2 years prior)	25 mm (SoV)	Labetalol
**Patient 2**	33	29	I	HIC	G1P1	-	-	Negative	Positive	Postpartum hemorrhage (4 years prior)	-	-
**Patient 3**	27	25	I	HIC	G0P0	-	Deletion-insertion C.2553DELTINSAACAGACAGA	Negative	Positive	-	25 mm (SoV)	-
**Patient 4**	31	22	I	HIC	G0P0	Cardiological	Deletion-insertion C.3653_3686DELINSAATC	Negative	Positive	-	29 mm (AA + SoV)	Metoprolol
**Patient 5**	28	21	I	HIC	G1P1	-	Substitution C.1105G > C (P.GLY369ARG)	Negative	Positive	Antepartum hypertension	25 mm (AA)	Aspirin
**Patient 6**	28	30	I	HIC	G1P1	-	Substitution C.2356G > A (P.GLY786ARG)	Negative	Negative	Pre-eclampsia + preterm labour	-	-

AA, ascending aorta; HIC, high income country; LMIC, low-middle income country; NYHA, New York Heart Association; PV, pathogenic variant; SoV, sinus of Valsalva; SVEF, systemic ventricular ejection fraction.

**Table 2:** Detailed information during and after pregnancy of all patients.

	Max. diameter during	Medication during pregnancy	Cardiac complications	Obstetric complications	Delivery	Birth weight child (grams)	Child Apgar score at 1 min.	Complications child	Max. diameter after
**Patient 1**	30 mm (SoV)	Labetalol	-	-	CS (36 weeks)	2730 (50th percentile)	8	Pneumo-thorax	30 mm (SoV)
**Patient 2**	29 mm (AA)	-	-	Threatened preterm labour + cervical shortening (27 weeks)	Vaginal (38 weeks)	2940 (25th percentile)	9	-	28 mm (AA)
**Patient 3**	31 mm (AA)	-	TE event in calf vein pp	1.5L blood loss during delivery	CS (35 weeks)	2320 (25th percentile)	9	-	30 mm (AA)
**Patient 4**	33 (SoV)	Metoprolol	-	Vaginal bleeding (18 weeks) + PPROM with contractions	Emergency CS (36 weeks)	3380 (95th percentile)	9	-	34 (SoV)
**Patient 5**	-	Aspirin (started after vertebral artery dissection)	Bilateral vertebral artery dissection (separate points in time)	Threatened miscarriage (14 weeks)	-	-	-	Miscarriage (24 weeks)	22 mm (AA)
**Patient 6**	-	-	-	Pre-eclampsia	CS (37 weeks)	Unknown	Unknown	-	-

AA, ascending aorta; CS, caesarean section; pp, postpartum; PPROM, preterm prelabour rupture pf membranes; SoV, sinus of Valsalva; TE, thromboembolic.

Patient 1 was a 32-year-old nulliparous woman with previous therapeutic abortion and a miscarriage without aortic dilatation pre-pregnancy (maximum diameter of 25 mm at the sinus of Valsalva (SoV) and 24 mm at the ascending aorta (AA)). At 33 weeks gestational age (GA), she was admitted to the hospital, because her echocardiogram showed an increase in AA dimension of 3 mm. A magnetic resonance angiogram was performed and showed only 1 mm increase. At 36 weeks GA, she delivered by planned caesarean section (CS) for cardiac indication. The infant experienced transient respiratory distress due to a pneumothorax requiring an intercostal catheter. There were no further complications, and she breastfed her infant for 30 weeks. Imaging data was available at six different time points. For the SoV, sino-tubular junction (STJ), AA and aortic arch a slight upward trend was observed (Fig. 1).

Patient 2 was a 33-year-old multiparous woman. She declined genetic testing, but was clinically diagnosed by the clinical geneticist based on symptoms and familial context. She had a relative with genetically confirmed vEDS. No cardiac complications occurred during pregnancy. She did experience threatened preterm labour and cervical shortening at 27 weeks GA. At 39 weeks GA, she was induced and delivered vaginally with epidural anesthesia. No complications occurred during delivery and postpartum. Imaging data of the annulus, SoV and AA at two time point during pregnancy and once at eight weeks postpartum showed slight enlargement at the annulus and AA (Fig. 2). No aortic measurements were available prior to pregnancy.

Patient 3 was a 27-year-old nulliparous woman. Her echocardiogram two years prior to this pregnancy showed a maximum diameter of 25 mm at the SoV. She was electively admitted to the hospital at 34 weeks GA for fetal pulmonary maturation. She did not experience any cardiac complications during pregnancy. Planned delivery at 34 + 6 weeks GA via CS was complicated by 1.5 L of blood loss. One week after delivery, she developed a deep venous thrombosis in the calf muscle vein, which was treated conservatively. After delivery, she breastfed her infant for two months, and no further complications occurred. Measurements of the SoV were available from before, during and after pregnancy. The measurements during and after pregnancy showed no dilatations of the aorta (Fig. 3).

Patient 4 was a 31-year-old nulliparous woman. Her AA and SoV were both 29 mm two years prior to this pregnancy. At 18 weeks GA, she developed transient vaginal bleeding that did not require treatment. At 36 + 3 weeks GA, she delivered by emergency CS due to PPROM with contractions. There were no further complications. Imaging data were available for the SoV, AA, aortic arch and descending aorta at six time points. For all segments, there was a minor increase in diameter comparing before and after pregnancy (Fig. 4).

Patient 5 was a 28-year-old multiparous woman. During a previous pregnancy, she had developed hypertension. During this pregnancy, she had a threatened miscarriage at 14 weeks GA. At 24 weeks, she experienced a stillbirth due to unknown reason, complicated by vaginal bleeding three days later. Limited imaging data were available. No serial aortic measurements were performed during pregnancy, and only the AA was measured more than once (25mm one year prior to pregnancy and 22mm one year after pregnancy).

Patient 6 was a 28-year-old multiparous woman. During a previous pregnancy, she developed pre-eclampsia and the infant was born preterm at 33 weeks GA.During this pregnancy, she also developed pre-eclampsia and delivered via planned CS at 37 weeks GA. There were no complications postpartum. No imaging data was available for this patient.

### 3.1 Imaging data

Combining all measurements of women with more than two measurements (before and after), we found no significant aortic growth at the SoV (p = 0.768) or the AA (p = 0.293). Both segments measured 26 mm on average before pregnancy. After pregnancy, the SoV was on average 28.5 mm and the AA was 27.2 mm. Other segments (annulus, STJ, arch, DA) had too few measurements for statistical comparison.

## 4 Discussion

This case series is the first prospective observational study on pregnant women with vEDS, who had been diagnosed before pregnancy. Importantly, no maternal deaths occurred and no aortic dissections were recorded. These results are reassuring, but this cohort is small and half of these patients were multiparous posing selection bias as these women are known to tolerate pregnancy. Larger prospective studies are needed.

One (16.7%) fetal death occurred at 24 weeks *in utero*. Obstetric complications were common (80%). Four out of five (80%) patients delivered via CS, because of their vEDS diagnosis. There was no significant aortic growth and no aortic dilatation was found in any patients. However, in vEDS patients it is unknown whether aortic dilatation precedes dissection.

The current ESC pregnancy guidelines state that women with vEDS are classified as mWHO IV indicating they should be advised against pregnancy [13]. However, the advice is more nuanced as the overall survival rate is not altered by pregnancy. The American Heart Association (AHA) guidelines state that with normal vascular imaging the risk may be lower, and that with shared decision-making pregnancy can be an option [14]. Having children can be incredibly important for a woman and her partner. Now, we presented six cases of women known with vEDS who survived pregnancy without any vascular events and five out of six women delivered a healthy child. A recent systematic review, including 412 pregnancies of vEDS patients, showed a mortality rate of 5.7% and a major complications (vascular/uterine/intestinal rupture) rate of 5.4% [7]. Notably, a separate study constructed Kaplan-Meier survival curves of women with vEDS comparing ever-pregnant to never-pregnant. The curves were similar, suggesting that pregnancy does not seem to alter overall survival [8]. This is now also mentioned in the latest ESC guidelines. So, while complications do occur, the impact of pregnancy might be less negative than previously believed and more nuanced advice might be justifiable.

A variety of pathogenic variants have been found in the *COL3A1* gene of vEDS patients. A previous study found an association between the type of pathogenic variant and the phenotype [5]. The in-frame insertions-deletions from patients 3 and 4 were located in the collagen helical domain, which is associated with more severe disease according to the study, while patient 1, 5 and 6 had glycine substitutions which are also associated with a more severe phenotype. However, it has been reported that aortic complications specifically are more prevalent in patients carrying haplo-insufficient variants. None of these patients had this type of pathogenic variant. Nevertheless, genotyping the patients is important to improve pre-pregnancy advice. In this study, no data was collected on prenatal genetic testing of the fetus. This would be useful in practice considering the relation between fetal genetic status and PPROM [15].

Obstetric complications are relatively common in women with vEDS. However, a recent systematic review found that the premature delivery rate (7.3%) is similar to the global prematurity rate [16]. This review also found that most major complications happened postpartum after vaginal delivery and most maternal deaths occurred during delivery often due to vascular rupture [7]. In our cohort, three out of five (60%) live infants were delivered prematurely: one emergency CS due to PPROM with contractions and two planned CS at 35 and 36 weeks. Although partly iatrogenic, this relatively high rate of premature deliveries may be explained by heightened vigilance for complications related to earlier reports. The cumulative data suggest that complications are rare during gestation and thus, in the interest of the infant, delaying a planned CS until at least 37 weeks is advisable.

Studies in patients with other connective tissue disorders suggest that the aortic growth is enhanced during pregnancy [17]. This is the first study to address this matter in vEDS patients. Our analyses showed no significant growth of the aorta at the level of the SoV or AA. However, the imaging data from Patient 1 and Patient 4 separately show some growth at several segments. Patient 1 had an increase of 5mm at the SoV and an increase of 6mm at the aortic arch. Patient 4 did not experience a major increases.

Another important aspect is the use of preventative medication during pregnancy. The current ESC guidelines recommend the use of celiprolol during pregnancy in women with vEDS. This is based on a prospective randomised trial in French and Belgian non-pregnant vEDS patients, which concluded that celiprolol use was associated with reduced rates of aortic dissection [18]. The AHA also recommends beta-blocker therapy during pregnancy, although they did not specify which beta-blocker. In our cohort, no women were taking celiprolol, while two women used another type of beta-blocker (labetalol and metoprolol). More research on celiprolol use during pregnancy is warranted.

Until now, no data was available on breastfeeding in vEDS patients. Breastfeeding is considered important for women, so it is crucial to have data on safety. In our cohort, three patients had been breastfeeding and no complications occurred. These numbers are too small to draw any conclusions, but we did not find any evidence to discourage breastfeeding.

## 5 Conclusion

The risk of pregnancy in women known with vEDS pre-pregnancy seems lower than previously reported. However, obstetric complications occurred frequently, and pre-pregnancy counseling and genotyping remains crucial.

## CRediT authorship contribution statement

**P.N.J. Peters:** Writing – review & editing, Writing – original draft, Visualization, Software, Formal analysis. **J.A. van der Zande:** Writing – review & editing, Supervision, Investigation, Data curation. **S.K. Prakash:** Writing – review & editing, Data curation. **J. Harris:** Writing – review & editing, Data curation. **E. Troost:** Writing – review & editing, Data curation. **D. Tobler:** Writing – review & editing, Data curation. **B.J. Bouma:** Writing – review & editing, Data curation. **J.W. Roos-Hesselink:** Supervision, Project administration, Methodology, Funding acquisition, Conceptualization.

## Statement of consent

This study complies with the Declaration of Helsinki. Participating centres in the ROPAC managed the approvals of national or regional ethics committees or Institutional Review Boards. Informed consent was obtained from all patients.

## Funding

This work was supported by the 10.13039/501100000860European Society of Cardiology EURObservational Research Programme. Funding from “Zabawas Foundation” and “De Hoop Foundation” in addition to the support from EURObservational Research Programme is greatly acknowledged. Since the start of EURObservational Research Programme, the following companies have supported the programme: 10.13039/100011949Abbott Vascular Int. (2011-2021); 10.13039/100002429Amgen Cardiovascular (2009-2018); 10.13039/100004325AstraZeneca (2014-2021); 10.13039/100004326Bayer AG (2009-2018); 10.13039/100001003Boehringer Ingelheim (2009-2019); 10.13039/100008497Boston Scientific (2009-2012); The 10.13039/100002491Bristol Myers Squibb and 10.13039/100004319Pfizer Alliance (2011-2019); 10.13039/501100022274Daiichi Sankyo Europe GmbH (2011-2020); 10.13039/501100021202the Alliance 10.13039/501100022274Daiichi Sankyo Europe GmbH and 10.13039/100004312Eli Lilly and Company (2014-2017); 10.13039/100006520Edwards (2016-2019); Gedeon Richter Plc. (2014-2016); Menarini Int. Op. (2009-2012); MSD-Merck & Co. (2011-2014); Novartis Pharma AG (2014-2020); ResMed (2014-2016); Sanofi (2009-2011); Servier (2009-2021) and Vifor (2019-2022).

## Declaration of competing interest

The authors declare the following financial interests/personal relationships which may be considered as potential competing interests: Siddharth Prakash reports was provided by The University of Texas Health Science Center at Houston. If there are other authors, they declare that they have no known competing financial interests or personal relationships that could have appeared to influence the work reported in this paper other than JWR-H serving the IJCCHD Editorial Board but had no involvement in the handling of this paper.

## Appendices

### EORP Oversight Committee

**2014-2016**: R. Ferrari, IT (Chair); A. Alonso, ES; J. Bax, NL; C. Blomström-Lundqvist, SE; S. Gielen, DE; P. Lancellotti, BE; A.P. Maggioni, IT; N. Maniadakis, GR; F. Pinto, PT; F. Ruschitzka, CH; L. Tavazzi, IT; P. Vardas, GR; F. Weidinger, AT; U. Zeymer, DE. **2016-2018**: A. Vahanian, FR (Chair); A. Budaj, PL; N. Dagres, DE; N. Danchin, FR; V. Delgado, NL; J. Emberson, GB; O. Friberg, SE; C.P. Gale, GB; G. Heyndrickx, BE; B. Iung, FR; S. James, SE; A.P. Kappetein, NL; A.P. Maggioni, IT; N. Maniadakis, GR; K.V. Nagy, HU; G. Parati, IT; A-S. Petronio, IT; M. Pietila, FI; E. Prescott, DK; F. Ruschitzka, CH; F. Van de Werf, BE; F. Weidinger, AT; U. Zeymer, DE. **2018-2020**: C.P. Gale, GB (Chair); B. Beleslin, RS; A. Budaj, PL; O. Chioncel, RO; N. Dagres, DE; N. Danchin, FR; J. Emberson, GB; D. Erlinge, SE; M. Glikson, IL; A. Gray, GB; M. Kayikcioglu, TR; A.P. Maggioni, IT; K.V. Nagy, HU; A. Nedoshivin, RU; A-P. Petronio, IT; J.W. Roos-Hesselink, NL; L. Wallentin, SE; U. Zeymer, DE. **2020-2022**: B.A. Popescu, RO (Chair); D. Adlam, GB; A.L.P. Caforio, IT; D. Capodanno, IT; M. Dweck, GB; D. Erlinge, SE; M. Glikson, IL; J. Hausleiter, DE; B. Iung, FR; M. Kayikcioglu, TR; P. Ludman, GB; L. Lund, SE; A.P. Maggioni, IT; S. Matskeplishvili, RU; B. Meder, DE; K.V. Nagy, HU; A. Nedoshivin, RU; D. Neglia, IT; A.A. Pasquet, BE; J.W. Roos-Hesselink, NL; F.J. Rossello, ES; S.M. Shaheen, EG; A. Torbica, IT.

### Executive Committee

Jolien Roos-Hesselink, The Netherlands (Co-chair); Roger Hall, United Kingdom (Co-chair); William Parsonage, Australia; Werner Budts, Belgium; Julie de Backer, Belgium; Jasmin Grewal, Canada; Ariane Marelli, Canada; Guillaume Jondeau, France; Mark Johnson, United Kingdom; Catherine Otto, United States; Karen Sliwa, South Africa; Aldo Maggioni, Italy.

### Investigators

**Armenia:** *Yerevan:* K. Vardanyan, A. Melkonyan, H. Lachikyan, K. Hakobyan, M. Mazmanian, *Yerevan:* H. Hayrapetyan, A. Tavaracyan, H. Poghosyan, R. Hovhannisyan, S. Sahakyan, S. Martirosyan, **Australia:** *St Leonards:* J. Harris, **Belgium:** *Brussels:* A. Pasquet, *Brussels:* M. Morissens, T. Besse-Hammer, B. Dumoulin, *Gent:* J. De Backer, L. Campens, L. Demulier, M. De Hosson, *Leuven:* W. Budts, A. Van de Bruaene, A. Rampelberg, E. Troost, L. Roggen, P. De Meester, **Botswana:** *Gaborone:* J.C. Mwita, E. Tefera, L. Kontle, **Canada:** *Montreal:* A. Marelli, I. Malhamé, *Vancouver:* J. Grewal, M. Janzen, **Cuba:** *La Habana:* P.A. Román Rubio, R. Vasallo Peraza, G. Vázquez Hernández, J.E. Pérez Torga, Y. Gil Jiménez, M. Meluzá Martín, **Egypt:** *Cairo:* R. Almaleh, *Cairo:* G. Youssef, K. Sorour, **Ethiopia:** *Addis Ababa:* S. Abebe, D. Mekonnen, C. Fekadu, D. Yadeta, **France:** *Bron:* S. Dupuis-Girod, L. Delagrange, *Lille:* M. Richardson, L. Ghesquiere, O. Domanski, M. Gonzalez Estevez, Y. Ould Hamoud, S. Gautier, L. Marsili, *Marseille:* L. Bal-Theoleyre, S. Palazzolo, *Paris:* M. Ladouceur, *Paris:* G. Jondeau, A. Bourgeois Moine, L. Eliahou, O. Milleron, M. Tchitchinadze, *Toulouse:* Y. Dulac, C. Karsenty, N. Souletie, F. Bajanca, **Germany:** *Hamburg:* C. Rickers, S. Blankenberg, C. Sinning, C. Magnussen, E. Zengin, G. Mueller, R. Schnabel, Y. Von Kodolitsch, R. Kozlik-Feldmann, *Muenster:* H. Baumgartner, R. Schmidt, A. Hellige, A. Rietkötter, **Greece:** *Athens:* M. Spartalis, *Athens:* A.A. Frogoudaki, *Thessaloniki:* A. Arvanitaki, A. Baroutidou, G. Giannakoulas, C. Karvounis, **India:** *Chennai:* J.P. Gnanaraj, A.P. Steaphen, T. Ethirajan, K. Kannan, V. Subramanian, A. Surendran, J. Gnanasekaran, V. Natarajalingam, S. Balasubramani, **Iraq:** *Bagdad:* H. Ali Farhan, I.F. Yaseen, **Italy:** *Bologna:* E. Mariucci, C. Ciuca, *Massa:* F. Marchi, G. Benedetti, M. Baroni, P. Festa, A. Parlanti, *Naples:* G. Scognamiglio, F. Fusco, B. Sarubbi, *Trieste:* M. Merlo, B. D'Agata Mottolese, C. Carriere, G. Sinagra, M. Bobbo, F. Ramani, *Turin:* F.M. Comoglio, R. Bordese, A. Pagano, N. Montali, V. Donvito, C.A. Remolif, F. Petey, **Netherlands:** *Amsterdam:* B. Bouma, S. Chamuleau, D. Robbers-Visser, *Amsterdam:* T. Konings, H. Dronkert, *Breda:* D. Segers, *Nijmegen:* R. Van Kimmenade, *Utrecht:* H. Van Der Zwaan, G. Tjalling Sieswerda, A. Evers, T. Schaap, **Pakistan:** *Karachi:* K. Bano, H. Yasmeen, K. Amir, N. Patel, P. Akhter, R. Khan, A. Shakeel, S. Mahar, S. Habib, **Poland:** *Lodz:* M. Lelonek, *Warsaw:* P. Hoffman, M. Lipczynska, **Portugal:** *Lisbon:* L. De Sousa, V. Ferreira, T. Mano, M. Selas, R. Cruz Ferreira, **Russian Federation:** *Saint Petersburg:* E. Shlyakhto, O. Irtyuga, G. Sefieva, K. Malikov, T. Pervunina, U. Shadrina, **Saudi Arabia:** *Jeddah:* A. Kinsara, *Riyadh:* D. Galzerano, H. Al Sergani, W. Kurdi, N. Kholaif, O. Vriz, A. Alhamshari, A. Alsaigh, O. Ahmad, S. Alzaher, B. Alamro, **South Africa:** *Cape Town:* K. Sliwa, F. Jakoet-Bassier, **Spain:** *Barcelona:* L. Galian-Gay, A. Pijuan-Domenech, B. Miranda-Barrio, B. Gordon, **Sweden:** *Goteborg:* E. Furenäs, *Lund:* J. Hlebowicz, F. Wedlund, *Stockholm:* E. Nagy, E. Mattsson, M. Majczuk Sennstrom, P. Sörensson, *Uppsala:* C. Christersson, A. Lutvica, B. Jönelid, T. Achter, K. Junus, H. Gärdesten Wall, M. Andreasson, **Switzerland:** *Basel:* D. Tobler, *Genève:* J. Bouchardy, F. Brand, C. Blanche, *Lausanne:* J. Bouchardy, T. Rutz, F. Brand, **Türkiye:** *Ankara:* U. Canpolat, Y.Z. Sener, N. Ozer, *Istanbul:* E. Ayduk Gövdeli, Z. Bugra, B. Umman, P. Karaca Özer, D. Baykiz, *Istanbul:* D. Mutlu, H. Yalman, B. Kilickiran Avci, *Istanbul/turkey:* S. Catirli Enar, O. Batukan Esen, D. Oksen, **Ukraine:** *Kyiv:* V. Lazoryshynets, S. Siromakha, Y. Davydova, A. Limanska, I. Zinovchyk, V. Kravchenko, B. Kravchuk, O. Kravets, N. Volkova, O. Mazur, O. Beregovyi, **United Arab Emirates:** *Abu Dhabi:* B.T. Salih, W.A.R. Al Mahmeed, S. Wani, F.S. Mohamed Farook, G. Al Mansoori, **United States:** *Houston:* S. Prakash, R. Afifi, D. Milewicz, A. Cecchi, *Lexington, Kentucky:* G. Wells, D. Sparks, *Minneapolis, Mn:* W. Wagner, C. Bigelow, L. Colicchia, T. Jentink, M. Loichinger, R. Saxena, W. Wunderlich, C. Longtin, P. Klopper, R. Gobar, *New Haven:* J. Chou, K. Campbell, R. Elder, *New York:* D. Halpern, A. Hausvater, H. Reynolds, N. Bhalla, A. Small, J. Feinberg, P. Panday, *Phoenix:* J. Awerbach, J. Porche, S. Stack, *Portland:* L. Mcgrath, A. Khan, E. Pare, P. Woods, C. Broberg, K. Gibbins, K. Brookfield, *Stony Brook:* M. Al-Sadawi, A. Cove, N. Mann.

## References

[1] Pope F.M., Martin G.R., Lichtenstein J.R., Penttinen R., Gerson B., Rowe D.W., McKusick V.A. (1975). Patients with Ehlers-Danlos syndrome type IV lack type III collagen. Proc Natl Acad Sci U S A.

[2] Pepin M., Schwarze U., Superti-Furga A., Byers P.H. (2000). Clinical and genetic features of Ehlers-Danlos syndrome type IV, the vascular type. N Engl J Med.

[3] Byers P.H., Belmont J., Black J., De Backer J., Frank M., Jeunemaitre X. (2017). Diagnosis, natural history, and management in vascular Ehlers-Danlos syndrome. Am J Med Genet C Semin Med Genet.

[4] Pepin M.G., Schwarze U., Rice K.M., Liu M., Leistritz D., Byers P.H. (2014). Survival is affected by mutation type and molecular mechanism in vascular Ehlers-Danlos syndrome (EDS type IV). Genet Med.

[5] Frank M., Albuisson J., Ranque B., Golmard L., Mazzella J.M., Bal-Theoleyre L. (2015). The type of variants at the COL3A1 gene associates with the phenotype and severity of vascular Ehlers-Danlos syndrome. Eur J Hum Genet.

[6] Soma-Pillay P., Nelson-Piercy C., Tolppanen H., Mebazaa A. (2016). Physiological changes in pregnancy. Cardiovasc J Afr.

[7] Haem T., Benson B., Dernoncourt A., Gondry J., Schmidt J., Foulon A. (2024). Vascular Ehlers-Danlos syndrome and pregnancy: a systematic review. BJOG.

[8] Murray M.L., Pepin M., Peterson S., Byers P.H. (2014). Pregnancy-related deaths and complications in women with vascular Ehlers-Danlos syndrome. Genet Med.

[9] Slaoui A., Mahtate M., Lazhar H., Lakhdar A., Baydada A., Kharbach A. (2022). Spontaneous uterine rupture revealing vascular Ehlers-Danlos syndrome: an uncommon case report. Int J Surg Case Rep.

[10] Hutt E., Santos-Martins C., Aguilera J., Wierup P., Kalahasti V., Tan C. (2020). A 27-Year-Old woman with postpartum Papillary muscle rupture. JACC Case Rep.

[11] Koitabashi N., Yamaguchi T., Fukui D., Nakano T., Umeyama A., Toda K. (2018). Peripartum iliac arterial aneurysm and rupture in a patient with vascular Ehlers-Danlos syndrome diagnosed by next-generation sequencing. Int Heart J.

[12] Cereda A.F., Canova P.A., Soriano F.S. (2017). Spontaneous coronary artery dissection after pregnancy as first manifestation of a vascular Ehlers-Danlos syndrome. J Invasive Cardiol.

[13] De Backer J., Haugaa K.H., Hasselberg N.E., de Hosson M., Brida M., Castelletti S. (2025). 2025 ESC guidelines for the management of cardiovascular disease and pregnancy. Eur Heart J.

[14] Isselbacher E.M., Preventza O., Hamilton Black J., Augoustides J.G., Beck A.W., Bolen M.A. (2022). 2022 ACC/AHA guideline for the diagnosis and management of aortic disease: a report of the American heart association/American college of cardiology joint committee on clinical practice guidelines. Circulation.

[15] Underhill L.A., Barbarita C., Collis S., Tucker R., Lechner B.E. (2022). Association of maternal versus fetal Ehlers-Danlos syndrome status with poor pregnancy outcomes. Reprod Sci.

[16] Ohuma E.O., Moller A.B., Bradley E., Chakwera S., Hussain-Alkhateeb L., Lewin A. (2023). National, regional, and global estimates of preterm birth in 2020, with trends from 2010: a systematic analysis. Lancet.

[17] Donnelly R.T., Pinto N.M., Kocolas I., Yetman A.T. (2012). The immediate and long-term impact of pregnancy on aortic growth rate and mortality in women with Marfan syndrome. J Am Coll Cardiol.

[18] Ong K.T., Perdu J., De Backer J., Bozec E., Collignon P., Emmerich J. (2010). Effect of celiprolol on prevention of cardiovascular events in vascular Ehlers-Danlos syndrome: a prospective randomised, open, blinded-endpoints trial. Lancet.

